# A mixture of 86% of CO_2_, 10% of N_2_O, and 4% of oxygen permits laparoscopy under local anesthesia: a pilot study

**DOI:** 10.1007/s10397-014-0872-2

**Published:** 2014-12-14

**Authors:** Philippe R. Koninckx, Jasper Verguts, Roberta Corona, Leila Adamyan, Ivo Brosens

**Affiliations:** 1 UZ Gasthuisberg, Department of Obstetrics and Gynecology, KULeuven, 3000 Leuven, Belgium; 2 Gruppo Italo Belga, Vuilenbos 2, 3360 Bierbeek, Belgium; 3 Centre for Reproductive Medicine, Free University Brussels, 101 Laarbeeklaan, 1090 Brussels, Belgium; 4 Department of Obstetrics and Gynecology, Jessa Hospital, Hasselt, Belgium; 5 Department of Reproductive Medicine and Surgery, Moscow state University of Medicine and Dentistry, Moscow, Russia

**Keywords:** Anesthesia, Conditioning, Gas, Laparoscopy, Pain, Pneumoperitoneum

## Abstract

The aim of this study is to verify that 10 % of N_2_O in CO_2_ sufficiently reduces pain to permit laparoscopy under local anesthesia. In nine patients undergoing laparoscopy under local anesthesia for tubal sterilization, a mixture of 86 % of CO_2_, 10 % of N_2_O, and 4 % of oxygen (the Gas Mixture) was used for the pneumoperitoneum. For CO2, N_2_O, and for the Gas Mixture, the pain when blowing over the tongue tip and the pH changes of saline and Hartmann’s solution were estimated. In all nine patients, discomfort was minimal and the intervention was well tolerated, similar to 100 % N_2_O. Tongue tip pain (*n* = 15), on VAS scale, was lower with 86 % CO_2_ + 10 % N_2_O + 4 % O_2_ (2.4 ± 1.4, *P* = 0.005) and much lower with 100 % N_2_O (0.3 ± 0.6, *P* < 0.0007) than with pure CO_2_ (3.6 ± 1.7). The pH of saline (*n* = 5) decreased from 7.00 ± 0.07 to 4.18 ± 0.04 (*P* = 0.001), 6.98 ± 0.08 (NS), and 4.28 ± 0.04 (*P* = 0.01) with 100 % CO_2_, 100 % N_2_O and the Gas Mixture_,_ respectively. The pH of Hartmann’s solution (*n* = 5) decreased similarly from 7.00 ± 0.07 to 5.18 ± 0.04 (*P* = 0.01), 7.02 ± 0.19 (NS), and 5.3 ± 0.4 (*P* = 0.01), respectively. These data demonstrate that a mixture with 10 % of N_2_O and 4 % of O_2_ in CO_2_ permits laparoscopy under local anesthesia. This result cannot be explained by direct irritation estimated by tongue tip pain or by pH changes.

## Introduction

Laparoscopy under local anesthesia has never become popular notwithstanding the advantages of a short hospital stay without general anesthesia. Following the report in 1976 of salpingectomies for tubal sterilization using umbilical local anesthesia, slight sedation, and pure N_2_O for the pneumoperitoneum [1], a Yoon ring tubal sterilization program under local anesthesia was started in 1976 in Leuven [2]. Although pure N_2_O is less painful than CO_2_ for the pneumoperitoneum [3–6], laparoscopy under local anesthesia using CO_2_ pneumoperitoneum can be performed albeit with stronger sedation and/or microlaparoscopy [7–14].

That 100 % N_2_O for the pneumoperitoneum causes less pain after surgery than 100 % CO_2_, was demonstrated in randomized controlled trials [15, 16]. The mechanism through which a N_2_O pneumoperitoneum causes little pain in comparison with CO_2_ was believed to be a consequence of the absence of the irritation of CO_2_. The use of other inert gases as helium and argon under local anesthesia was never reported to the best of our knowledge. The use of N_2_O for the pneumoperitoneum is safe since the solubility of N_2_O in blood and the exchange capacity in the lungs is comparable or better than CO_2_. N_2_O, in addition, avoids the metabolic effects of CO_2_ resorption [17–21]. Nevertheless, the clinical use of N_2_O for inducing the pneumoperitoneum during operative laparoscopy never became popular because of the explosion risk when using electrosurgery at concentrations of N_2_O higher than 29 % [22, 23].

We recently demonstrated in our laparoscopic mouse model [24, 25] that the effect of as little as 5 % of N_2_O in CO_2_ had a similar effect in reducing postoperative adhesions as pure N_2_O. In a randomized controlled trial (RCT) in the human [26], we subsequently demonstrated the virtual absence of adhesions and a strong decrease in pain following full-conditioning during surgery (i.e., 10 % of N_2_O and 4 % of O_2_ in CO_2_ for the pneumoperitoneum, cooling of the peritoneal cavity to 30 °C, and absence of desiccation) and a barrier at the end of surgery in patients undergoing deep endometriosis excision.

We therefore planned an observational trial to test the hypothesis that 10 % of N_2_O in CO_2_ would reduce pain and permit laparoscopy under local anesthesia similar as 100 % of N_2_O does.

## Materials and methods

### Tubal sterilization under local anesthesia

Since 1976, tubal sterilization under local anesthesia using 100 % N_2_O for the pneumoperitoneum has been a routine procedure in the university hospitals of the Catholic University of Leuven (KULeuven) [2]. Following local anesthesia of the umbilicus with 10 ml of 2 % xylocaine, the pneumoperitoneum was induced with pure N_2_O using a water valve limiting the pneumoperitoneum pressure to 15 mm of Hg, while all extra gas was permitted to escape freely [27] An insufflator CE marked to be used with N_2_O indeed did not exist. The umbilical trocar was inserted with active pressure of the patient to distend the abdomen, thus increasing the distance between the peritoneal wall and the large vessels and the safety of insertion. Subsequently, using an operative laparoscope (initially the 12-mm KLI, USA single incision applicator; later the Storz AG, Tüttlingen Germany, operative laparoscope), 10 ml of an anesthetic gel (xylocaine gel, Astra Zeneca) was applied over the oviducts. Initially, only Yoon rings were applied; more recently, the department decided to use Filshie clips. The entire procedure of tubal sterilization under local anesthesia rarely exceeded 5 min. A short duration indeed is crucial for acceptability by the patient who becomes increasingly nervous when the procedure takes longer or when there is any sign of nonconfidence by the surgeon. Sedation before surgery consisted initially of Dipidolor (Janssens, Belgium). Later sedation was omitted if the patient was not too anxious. This technique had been used for 30 years in over 1000 patients without a single major complication and without a failure. Although the technique was almost systematically used in the late 1970s and early 1980s, general anesthesia became subsequently predominantly used in the department since the necessity of a short procedure and of a confident surgeon conflicted with the necessity of training the registrars.

### Observational trial using 86% CO_2_, 10% of N_2_O, and 4% of O_2_ (the Gas Mixture) for the pneumoperitoneum

In order to evaluate whether this mixture would be sufficient to permit laparoscopy under local anesthesia, this mixture was used instead of 100 % N_2_O in all nine patients scheduled for laparoscopic sterilization under local anesthesia by PK from September 30, 2010, till September 30, 2011. The age of the women included ranged from 31 to 46 years and their weight from 61 to 85 kg.

Informed consent was obtained prior to the procedure with the explicit agreement that in case of pain, a general anesthesia would be performed immediately. All procedures were in accordance with the ethical standards of the responsible committee on human experimentation (institutional and national) and with the Helsinki Declaration of 1975, as revised in 2008. IRB approval had been obtained in September 2010 for the use of CO_2_ with 10 % of N_2_O and 4 % of O_2_ for the pneumoperitoneum, e.g., for the randomized controlled trial on postoperative pain and adhesion formation [26].

The primary aim of the trial was to assess feasibility of the procedure without discomfort of the patient.

### Tongue tip pain and pH

In order to measure the irritation by 100 % CO_2_, 86 % CO_2_ + 10 % N_2_O + 4 % O_2,_ and 100 % N_2_O in 15 healthy volunteers (registrars between 23 and 31 years old), the severity of pain was assessed by a visual analog scale after directing through a Pasteur pipette a flow of 2 L/min to the tongue at 1 cm distance for 30 s. Also, the pH of saline and of Hartmann’s solution was measured following equilibration with the three gases for 5 min.

### Statistics

Means and standard deviations are given. For the pain dataset, overall statistical significance was calculated using Friedman’s test (nonparametric paired ANOVA), while differences between groups was calculated by Wilcoxon matched pairs test. For the pH data, overall statistical significance was calculated using Kruskal–Wallis test (nonparametric unpaired ANOVA), while differences between groups was calculated by Mann–Whitney test. Analysis was done with GraphPad Prism (GraphPad software).

## Results

In all nine patients, little or no pain was experienced during the induction of the pneumoperitoneum, and the procedures were comparable with previous interventions using 100 % N_2_O for the pneumoperitoneum. All nine patients were discharged a few hours after the intervention and could return to their normal activity within a few days.

The tongue tip pain (*n* = 15) on VAS scale (Friedman <0.0001), was lower with the Gas Mixture (2.4 ± 1.4, *P* = 0.005) and with 100 % N_2_O (0.3 ± 0.6, *P* < 0.0007) than with pure CO_2_ (3.6 ± 1.7). It was lower with N_2_O than with the Gas Mixture (*P* < 0.0007). The pH of saline (*n* = 5) decreased (Kruskal–Wallis *P* = 0.007) from 7.00 ± 0.07 to 4.18 ± 0.04 (*P* = 0.001), to 6.98 ± 0.08 (NS) and to 4.28 ± 0.04 (*P* = 0.01, NS versus CO_2_) with 100 % CO_2_, 100 % N_2_O and the Gas Mixture. The pH of Hartmann’s solution (*n* = 5) decreased (Kruskal–Wallis *P* = 0.0008) similarly from 7.00 ± 0.07 to 5.18 ± 0.04 (*P* = 0.01), to 7.02 ± 0.19 (NS), and to 5.3 ± 0.4 (*P* = 0.01, NS versus CO2), respectively.

## Discussion

Although the numbers are small, these data demonstrate that the use of 10 % of N_2_O and 4 % of O_2_ in CO_2_ for the pneumoperitoneum causes little peritoneal pain and permits laparoscopy under local anesthesia comparable to 100 % N_2_O. Feasibility of laparoscopic sterilization under local anesthesia is close to a black and white result. If the procedure is short and the surgeon is confident and keeps intermittently eye contact with the patient, with or without showing the surgery on the screen, the procedure is uneventful and the patient tells afterwards that discomfort was minimal. If however, the patient looses confidence for whatever reason, e.g., because of pain, because the procedure takes longer than 5 to 7 min, because the surgeon starts sweating or displays any other signs of nervousness, because of a higher insufflation pressure, or more Trendelenburg positioning, the anxiety of the patient increases rapidly and the procedure becomes difficult and stressful for both, if not impossible. The patient afterwards describes this pain as anxiety. The procedure thus requires an experienced and fast laparoscopic surgeon. This was the main reason that laparoscopic sterilization under local anesthesia proved difficult to introduce as a routine while most of the registrars stopped to use the procedure after one minor but for them stressful incident with anxious patient.

The use of 10 % of N_2_O has a major advantage in comparison with 100 % N_2_O since the explosion risk is absent at a concentration below 29 % of N_2_O, thus permitting eventual electrosurgery, e.g., to coagulate a bleeding. Another theoretical advantage is the reduced operating theater contamination in case of gas leaks and poor ventilation [28].

A mixture of 10 % of N_2_O and 4 % of O_2_ in CO_2_ was chosen for the following reasons. Although in mice it had been demonstrated that 5 % of N_2_O in CO_2_ was as effective as 100 % of N_2_O in reducing the acute inflammatory reaction and the subsequent enhanced adhesion formation caused by pure CO_2_ [29], we preferred for this human experiment to use 10 % of N_2_O since it remains far below the critical concentration of 29 % when explosions might occur. Although in the mouse model, no additive effect of 4 % oxygen could be demonstrated when 5 % of N_2_O or more was used [25], we preferred to use also 4 % of O_2_ for this exploratory trial since 4 % of oxygen when used alone had a small effect on postoperative pain in women [30].

The mechanism by which 100 % N_2_O and 10 % N_2_O + 4 % O_2_ in CO_2_ cause much less pain than CO_2_ during pneumoperitoneum is unclear. In our hands, insufflation with CO_2_, as attempted during the 1980s, immediately causes a sharp pain and the procedure had to be interrupted. In order to understand the mechanism of reduced pain by using 100 % N_2_O or the Gas Mixture, we measured the tongue tip pain and the pH changes caused by the different gases. CO_2_ induces strong irritation of the tongue; 100 % N_2_O was much less painful and the Gas Mixture with 10 % N_2_O only slightly reduced the tongue tip pain. The effect on the tongue tip pain is comparable with the pH changes which are very pronounced with CO_2_, almost inexistent with 100 % N_2_O whereas the Gas Mixture decreased pH only slightly less than 100 % CO_2_. Somatic pain of the tongue thus seems related to the irritative effect of CO_2_ and the changes in pH. The mechanisms of visceral pain of the peritoneum are known to be different [31], and we do not have an explanation why 10 % of N_2_O seems to be as effective as 100 % in reducing pain during laparoscopy under local anesthesia. This, however, is consistent with the effect of 100 % and 10 % N_2_O upon adhesion formation and upon postoperative pain [26] and suggests an unknown drug-like effect of N_2_O upon visceral pain.

It is unclear whether in the human that the addition of 4 % of oxygen has an additive pain-reducing effect. Unfortunately, we realize that the demonstration of an additive effect of 4 % of O_2_ will require large series to reach statistical significance, while clinically not important. The same holds true for the use of 5 % of N_2_O instead of 10 %. The only theoretical advantage of not using 4 % of O_2_ is the lower risk of gas embolism since the solubility of O_2_ in the blood is very low. With 4 % of O_2_, the risk however is considered close to nonexistent.

In conclusion, the use of 10 % of N_2_O in CO_2_ is a preferred alternative to pure N_2_O for laparoscopy under local anesthesia because of the absence of explosion risk by concentrations of N_2_O lower than 29 %, thus permitting electrosurgery when needed. This mixture moreover is extremely safe since N_2_O has an even higher solubility in water and exchange capacity in the lungs than CO_2_. The effect cannot be explained by pH changes or a direct irritation as observed on the tongue.
